# Physiological consequences of biologic state and habitat dynamics on the critically endangered Yangtze finless porpoises (*Neophocaena asiaeorientalis *ssp.* asiaeorientalis*) dwelling in the wild and semi-natural environment

**DOI:** 10.1093/conphys/coy072

**Published:** 2018-12-18

**Authors:** Ghulam Nabi, Yujiang Hao, Todd R Robeck, Zheng Jinsong, Ding Wang

**Affiliations:** 1 Institute of Hydrobiology, the Chinese Academy of Sciences, Wuhan, Hubei Province, People’s Republic of China; 2 University of Chinese Academy of Sciences, 19 Yuquan Road, Shijingshan District, Beijing, People’s Republic of China; 3 SeaWorld Parks and Entertainment, 9205 South Park Center Loop, Suite 400, Orlando, FL, USA

**Keywords:** biochemistry, Cetacean, critically endangered, habitat dynamics, hepatic profile, lipid profile, Yangtze Finless Porpoise

## Abstract

The objectives of this study were to investigate the effects of habitat and biological state on the physiology of critically endangered wild and semi-natural Yangtze Finless Porpoises (YFPs; *Neophocaena asiaeorientalis *ssp. *asiaeorientalis*) by measuring and comparing serum biochemical parameters. A total of 168 YFPs were sampled, 68 living in the semi-natural (Tian-E-Zhou Oxbow) and 98 living in the wild (Poyang Lake, PL) environment. The YFPs in the Tian-E-Zhou Oxbow were sampled from 2002 to 2015 and in the PL from 2009 to 2017. Each population was divided into Juvenile Male, Juvenile Female, Adult Male, Pregnant and Lactating Female life history categories. Overall, with location, 19/33 of the analytes and with season 18/33 of the analytes were significantly different. Similarly, within each location, 15/33 of the analytes changed with time in PL while only 8/33 changed with time in Tian-E-Zhou Oxbow, respectively. Finally, 15/33 of the analytes demonstrated significant differences between the different age and sex groups of animals. In our study, a significant variation, as well as an increasing and decreasing pattern of several parameters in both populations, suggest a worsening ecological environment of both habitats. This study will help in health assessment, improving conservation and management practices, a crucial requisite for biodiversity conservation.

## Teaser

This research describes the effect of age, sex, reproductive status and habitat location on serum biochemical analytes in the critically endangered Yangtze finless porpoise (*Neophocaena asiaeorientalis *ssp.* asiaeorientalis*). Results highlight conservation relevant effects of biologic stages and suggests that without proper management habitat degradation may adversely affect their long-term sustainability.

## Introduction

Effects of climate change and population growth on global freshwater reserves are increasing to such an extent that in some locations this valuable resource is shrinking, and in some instances, may disappear (Coe and Foley, 2001; Oren *et al.*, 2010). Poyang Lake (PL), the largest freshwater lake in China and an extension of the Yangtze River, is an important habitat for critically endangered Yangtze finless porpoises (YFPs,* Neophocaena phocaenoides asiaorientalis*; Wang *et al.*, 2013). In addition to significant water loss from a prolonged drought (Mei *et al.*, 2015; Zhang *et al.*, 2016), this crucial habitat is under increasing anthropogenic pressures in the form of sand dredging (Li, 2008), vessel trafficking (Zhang, 2007) and water quality degradation due to Yangtze River watershed alterations (Wu *et al.*, 2004; Mueller *et al.*, 2008). In addition to direct impacts on water quality, habitat fragmentation from these alterations may only increase with future hydroelectric power plant projects (Jiangxi Water Conservancy, 2010). Such changes in the Yangtze River are believed to have played a part in the rapid decrease of the YFP population numbers, which have been declining at the average rate of 13.73% per annum (Mei *et al.*, 2014). These declines have resulted in the latest estimated total YFP population numbers to be around 1000 individuals, with almost half (~450) of the living population in PL (Mei *et al.*, 2014). With the current population trajectory, the YFP may soon join the baiji (*Lipotes vexillifer*), a previous co-inhabitant of the Yangtze River, in extinction (Turvey *et al.*, 2007).

Worldwide deleterious alterations in habitat quality are believed to be the main cause of almost 40% of all mammalian species experiencing severe population declines (Ceballos *et al.*, 2017; Johnson *et al.*, 2017) with freshwater cetaceans, or river dolphins, appearing to be extremely vulnerable toward these pressures (Turvey *et al.*, 2010; He *et al.*, 2017). Habitat destruction in the biodiversity-rich area around the globe can cause mass extinction, especially for threatened and endemic species (Brooks *et al.*, 2002). Therefore, determining current physiological characteristics of individuals within an at-risk population provides baselines for future investigations on the potential effect of anthropogenic activities on their health and well-being (Schwacke *et al.*, 2009). It is well accepted that habitat quality effects body fitness and physiological functions of the animals living within these locations (Pulliam, 1988; Huey, 1991; Carey, 2005). Therefore, monitoring of physiologic traits can help us to understand and predict organismal and population responses to environmental change and stressors, cause-effect relationships, and specificity of management techniques (Christine *et al.*, 2016).

While the population of YFP in PL represents almost half of the remaining animals living within their historic range, an experimental conservation strategy involving relocation of a small number of animals into a natural *ex situ* reserve called the Tian-E-Zhou Oxbow (TZO) began in 1990. The use of this reserve, a habitat removed from many of the detrimental anthropogenic activity faced by *in situ* YFP, if successful over the long-term, would serve as a model for the identification of other natural habitats which could provide shelter for relocated YFP until the natural habitat of the Yangtze River could be sufficiently reclaimed for animal reintroduction. While the use of *ex situ* populations as a conservation strategy has been successfully implemented in terrestrial mammals, it represents the first application of this model toward a cetacean (O’Brien and Robeck, 2010). The initial success of the *ex situ* TZO population has been demonstrated by its increased from a net introduction of five animals from the wild since 1990 to a total of 25 in 2010. From 2010 to 2015, due to successful animal breeding, the population exceeds to more than 60 individuals with a net 108% increase in the population (Wang, 2009; 2015). This growing *ex situ* population, which has been living in a unique environment, may serve as a physiologic control for changes that may have occurred in wild populations of the Yangtze River, Poyang and Dongting Lake system. However, until recently (Nabi *et al.*, 2017a), most efforts at evaluating the health of the TZO *ex situ* population has focused on their reproductive health and resource management (Wu *et al.*, 2010; Zhao *et al.*, 2010; Zeng *et al.*, 2018). As a result, no information has been published examining the effects of their habitat relocation on their physiology over time. Therefore, the primary objective of our study was to determine if habitat, while controlling for season, had an adverse effect on the YFP health as indicated by differences in serum biochemical parameters within YFP between each location (PL vs. TZO). Secondarily, to determine if indirect evidence exists that habitats have degraded by evaluating changes in YFP serum biochemical profiles within each location over time. And finally, if changes existed between locations, to detect during what age (immature or mature), sex or physiologic state (pregnancy or lactation) they were occurring. The results of this study will help determine the efficacy of establishing *ex situ* natural reserves and provide evidence for or against the continued expansion of this conservation strategy.

## Materials and methods

### Animal ethics

The protocols for animal collection and handling were approved under Chinese law and guidelines for wildlife use by the Ministry of Agriculture of China. The Research Ethics Committee of Institute of Hydrobiology, the Chinese Academy of Science reviewed and approved the blood sampling and handling procedures.

### Study design

A totals of 168 animals were sampled from TZO (*n* = 70) and PL (*n* = 98). Information about animals and sampling dates are summarized in Tables 1 and 2. Data collected in 2002, 2003 and 2015 in the TZO and 2009 and 2015 in the PL have been previously reported (Nabi *et al.*, 2017a, 2018). Animals were grouped based on total body length (Gao and Zhou, 1993) and sex as follows: Juvenile male (JM < 138 cm), adult male (AM > 138 cm), juvenile female (JF < 138 cm) and adult females (AF > 138 cm, Table 1). Adult females were further classified as non-pregnant, lactating females (L) and pregnant non-lactating females (P). No non-pregnant, non-lactating females were sampled. Lactating females were identified based on the presence of milk in the mammary gland and pregnant females (PF) were identified by ultrasonography (LOGIQ Book XP, New York, America) of the reproductive tract.

**Table 1:** Basic information of the studied animals in Tian-E-Zhou Oxbow.

Status	ID	BL (cm)	BW (Kg)	Year/season	Status	ID	BL (cm)	BW (Kg)	Year/season
JM	02T-M1	114	23	2002	AM	06-T-M04	164	73.2	
JM	02T-M03	129	42	(Fall)	AM	06-T-M06	148	65.5	
JM	04-T-M04	123	34	2004 (summer)	AM	08-T-M02	161	68.75	2008 (spring)
JM	06-T-M07	134	42	2006	AM	08-T-M03	161	70.5	
JM	06-T-M08	137	49.4	(spring)	AM	08-T-M07	162	77	
JM	08-T-M01	133	38.6	2008	AM	08-T-M08	160	69	
JM	08-T-M04	133	39.5	(spring)	AM	08-T-M12	146	49	
JM	08-T-M06	114	29.6		AM	08-T-M13	142	53	
JM	08-T-M09	120	33.75		AM	10-T-M01	166	61.3	2010
JM	08-T-M10	134	44		AM	10-T-M02	163	69.9	(Fall)
JM	08-T-M11	118	34		AM	10-T-M03	158	70.6	
JM	10-T-M09	121	36.5	2010	AM	10-T-M04	165	68.8	
JM	10-T-M10	126	35	(Fall)	AM	10-T-M06	143	48.4	
JM	T15M07	125	31.4	2015	AM	10-T-M07	142	47.9	
JM	T15M18	133	35.2	(Fall)	AM	10-T-M08	145	47.6	
AM	02T-M01	152	53	2002	AM	T15M02	139	35.5	2015
AM	02T-M02	158	59.05	(Fall)	AM	T15M05	141	36.8	(winter)
AM	02T-M04	155	57.5		AM	T15M08	148	40.8	
AM	02T-M05	139	43		AM	T15M09	160	42	
AM	02T-M06	156	55.4		AM	T15M12	151	44.5	
AM	02T-M07	157	54.3		AM	T15M17	156	46.1	
AM	03T-M02	141	32.7	2003	P	08-T-F03	136	59	2008
AM	03T-M03	146	46.15	(Fall)	P	08-T-F05	140	53.25	(spring)
AM	03T-M04	158	57		P	08-T-F06	148	71.5	
AM	03T-M05	142	41.4		P	08-T-F07	152	63.75	
AM	03T-M06	149	50.15		P	08-T-F09	149	67.5	
AM	03T-M07	157	55.1		P	TEZ 18	149.5	51.1	2015
AM	03T-M08	154	55.8		P	TEZ 19	143	55.3	(winter)
AM	04-T-M01	159	59.35	2004	P	TEZ 21	147.5	53.4	
AM	04-T-M02	147	43.45	(Fall)	P	TEZ 25	139	45.8	
AM	04-T-M03	147	48.45		L	10-T-F01	148		2010
AM	04-T-M05	149	50.45		L	10-T-F03	140	51.2	(Fall)
AM	04-T-M06	149	48.7		L	10-T-F04	149	56	
AM	06-T-M01	149	50.2	2006	L	10-T-F09	140	58	
AM	06-T-M02	158	73.2	(spring)					
AM	06-T-M03	156	55.55

**Table 2:** Basic information of the studied animals in Poyang Lake.

Status	ID	BL (cm)	BW (Kg)	Year/season	Status	ID	BL (cm)	BW (Kg)	Year/season
JM	09PYM006	128	36.95	2009	AM	15PY-M09	160	57.2	
JM	09PYM008	127	36	(winter)	AM	15PY-M10	152	53.8	
JM	09PYM009	136	39.1		AM	15PY-M12	138	46.8	
JM	09PYM017	127	34.7		AM	15PY-M13	154	51.2	
JM	09PYM020	125	38.1		AM	17PYM15	145	49	2017
JM	10PYM03	127	35.1	2010	AM	17PYM06	150	45.1	(spring)
JM	10PYM04	114	30.9	(spring)	AM	17PYM14	142	49.7	
JM	11PYM03	124	39.2	2011	AM	17PYM07	146	47.7	
JM	11PYM04	124	35.3	(winter)	JF	10PYF05	128	34.8	2010
JM	11PYM06	137	45.4		JF	10PYF07	119	25.9	(spring)
JM	11PYM07	113	37.1		JF	11PYF02	127	39.3	2011
JM	11PYM08	113	35		JF	11PYF05	124	36	(winter)
JM	11PYM09	124	33.6		JF	11PYF07	118	37.3	
JM	11PYM11	116	38		JF	11PYF09	128	39	
JM	11PYM14	124	39.5		JF	11PYF10	112	32.5	
JM	11PYM15	116	39.4		JF	11PYF11	123	32.8	
JM	15PY-M01	122	36.8	2015	JF	11PYF19	119	33	
JM	15PY-M05	124	24	(spring)	JF	11PYF23	115	36	
JM	15PY-M07	112	31.8		JF	11PYF14	128		
JM	15PY-M11	129	37.6		JF	15PY-F04	115	34.7	2015
JM	15PY-M14	129	35.7		JF	15PY-F10	123	40.3	(spring)
JM	17PYM02	135.2	40.4	2017	JF	15PY-F12	127	45.7	
JM	17PYM10	128	39.2	(spring)	JF	15PY-F17	125	36.3	
JM	17PYM01	130	39.4		JF	17PYF14	122	33	2017
JM	17PYM16	132	41.9		JF	17PYF10	118	27.7	(spring)
AM	09PYM004	154	53	2009	P	09PYF002	145	62.9	2009
AM	09PYM007	150	53.5	(winter)	P	09PYF003	144	52.2	(winter)
AM	09PYM010	158	83.4		P	09PYF007	152		
AM	09PYM011	138	40.6		P	10PYF01	138	50.6	2010
AM	09PYM013	154	52.2		P	10PYF04	149	63.6	(spring)
AM	09PYM014	150	52.4		P	10PYF06	146	62.7	
AM	09PYM015	153	53.4		P	11PYF04	147	66.7	2011
AM	09PYM016	158	62.1		P	11PYF06	140	60.4	(winter)
AM	09PYM018	157	48.4		P	11PYF08	130	63.6	
AM	09PYM021	146	46.8		P	11PYF13	139	63	
AM	10PYM01	149	38.1	2010	P	11PYF15	129	55	
AM	10PYM05	152	47.4	(spring)	P	11PYF16	137	63.5	
AM	11PYM01	168	67.2	2011	P	11PYF18	148	61.1	
AM	11PYM02	166	74.1	(winter)	P	11PYF20	151	72.3	
AM	11PYM05	140	49		P	11PYF21	138	69.9	
AM	11PYM10	149	62		P	15PY-F22	138	67.5	2015
AM	11PYM13	146	48.6		P	15PY-F19	134	58.7	(spring)
AM	11PYM16	148	51.6		P	15PY-F18	148		
AM	11PYM17	150	59.1		P	15PY-F15	134	60.2	
AM	11PYM20	152	73.4		P	15PY-F05	147	72.1	
AM	15PY-M03	153	54.4	2015	P	17PYF11	160		2017
AM	15PY-M04	158	69.8	(spring)	P	17PYF04	148	62.1	(spring)
AM	15PY-M06	144	53.1		P	17PYF16	140	58.9	
AM	15PY-M08	145	49.8		P	17PYF02	162		

### Animal collection and blood sampling

Both in the PL and TZO, YFPs were captured by using the ‘sound chase and net capture’ method (Hua, 1987). The detailed information of animal chasing, catching, handling and release are explained in detail by Hao *et al.* (2009). Briefly, for each capture, animals were randomly selected within different geographical areas of the sampling locations for each collection attempt. The methodology during the capture event, the blood sampling procedure and the timing of blood collection were consistent for both populations. Ten ml of blood were drawn from the major vein of tail fluke aseptically using a disposable 10-ml syringe (Gemtier, G/Ø/ L: 21/0.7/31 mm, 201502, Shanghai, China). The blood was then transferred into serum separator and EDTA Vacutainer® tubes (BD Vacutainer, Becton Dickinson, Franklin Lakes, New Jersey, USA), and placed immediately on ice. After centrifugation (Eppendorf AG, 22332, Hamburg, Germany) at 1500 × g for 15 min, the obtained serum was then immediately transferred into cryotubes (Fisher Scientific, Pittsburgh, Pennsylvania, USA), and stored in a liquid nitrogen kettle for transportation to the laboratory for immediate analysis.

### Laboratory analyses

The liver function parameters; Indirect Bilirubin (I-BILI), Direct Bilirubin (D-BILI), Total Bilirubin (T-BILI), Total Bile Acid (TBA), Gamma-glutamyl Transferase (GGT), Alkaline Phosphatase (ALP), Aspartate amino Transferase (AST), Alaline amino Transferase (ALT), lipid profile; Ligh Density Lipoprotein cholesterol (LDL-c), High Density Lipoprotein cholesterol (HDL-c), Triglyceride (TG), Total Cholesterol (TC), enzymes; Lactate Dehydrogenase (LDH), Creatine Kinase (CK), Amylase (AMS), Electrolytes; (PO4^3−^, Ca^2+^, Cl^−^, Na^+^, K^+^, Mg^2+^, Fe^2+^) and other biochemical parameters such as Creatinine (Cr), Urea (UA), Blood Urea Nitrogen (BUN), Carbon Dioxide (CO_2_), Glucose (GLU), Globulin (GLB), Albumin (AlB) and Total Protein (TP) were investigated using a clinical auto-analyzer (Abbott Aeroset System). Before each assay, the auto-analyzer was calibrated.

### Statistical analysis

Based on sampling methodology, and for the analysis, animals were considered to have been randomly selected during each collection period and no animal was sampled more than once. Statistical analysis was performed by either STATA (version 14, Stata Corp LP, College Station, TX, USA) or Graph Pad Prism, *version 5*.*01* (*Graph Pad Software Inc*., San Diego, CA, USA). Prior to analysis, results for each analyte were evaluated for normality by the Shapiro–Wilk-test (STATA) and transformed as appropriate (natural log or sqrt). For the analysis, individual sample results for each analyte were coded for location (0: PL; 1: TZO), group (JM, JF, AM, PF, LF) and season (winter [W]: November through February; Spring [S]: March through May; Summer [Sm]: June through August; Fall [F]: September through October).

A maximum likelihood (ML) general linear model (GLM, identity link, Gaussian family) was used to determine if the dependent variable (analyte concentration) differed between locations (STATA) and between groups combined across both locations. Many of the analytes evaluated are known to be effective by season (Hall *et al.*, 2007; Macchi *et al.*, 2011; Nollens *et al.*, 2018); therefore, season was added as a covariate Post hoc marginal mean comparison between locations, groups and season were then preformed and data was presented as marginal mean and 95% confidence interval. To determine if changes had occurred for each dependent variable within each location over time (2002–15 for TZO and 2009–17 for PL) a linear regression was used with time as a continuous independent variable and group as a covariate (STATA).

An unpaired Student’s t-test was used to compare the analyte concentration within one group from PL to its respective group in TZO using Graph Pad Prism, *version 5*.*01* (*Graph Pad Software Inc*., San Diego, CA, USA). Results of the t-test between locations were presented as mean ± SEM. For all analyses, significance was defined as *P* ≤ 0.05.

## Results

**Figure 1:**
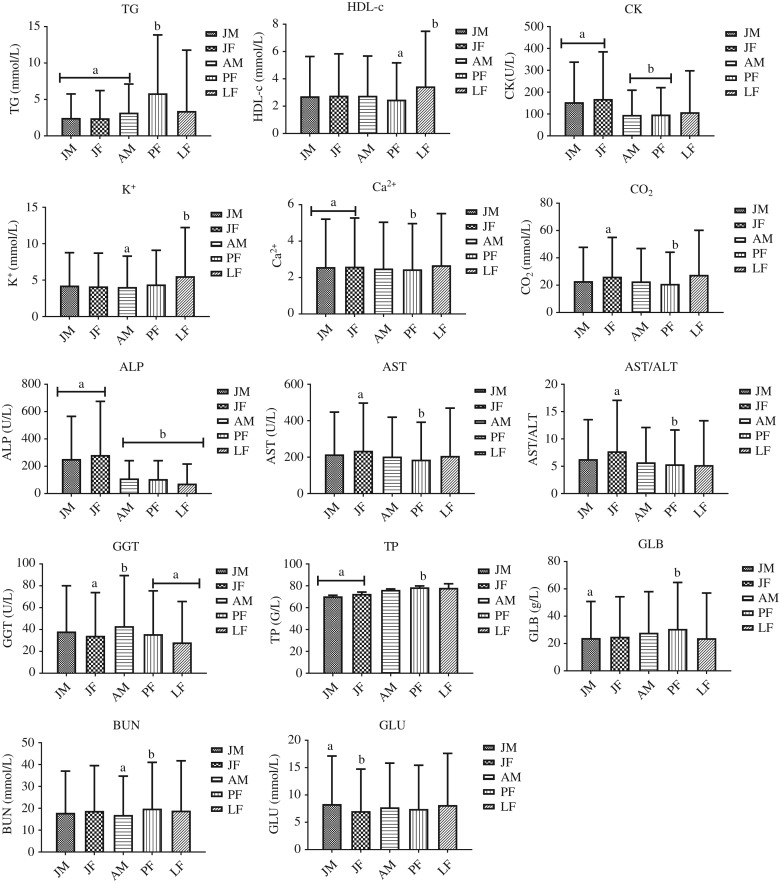
Overall marginal mean (±95% confidence interval) biochemical analyte comparisons in YFP from different groups (JM = juvenile male, JF = Juvenile female, AM = adult male, PF = non-lactating, pregnant female, LF = non-pregnant, lactating female). Marginal mean concentration for each analyte were controlled for variance due to location (TZO and PL) and season. Each biochemical parameter followed by an alternate letter was significantly different at *P* ≤ 0.05.

Overall, the results of the GLM analysis indicated that 58% (19 of 33) of the analytes were significantly different between locations, while, 55% (18 of 33) demonstrated significant seasonal effects (Table 3). However, a complete picture of the seasonal effects could not be determined due to the lack of sampling during the summer months. Across both locations, 46% (15/33) of the analytes demonstrated significant differences between the different age and sex groups of animals (Fig. 1). Finally, the linear regression results indicated that within each location, 46% (15 of 33) of the analytes changed over time in PL while only 24% (8 of 33) changed over time in TZO, respectively (Table 3).

**Table 3:** Effect of habitat (PL and TZO) and time (years, 2002 to 2015 for TZO and 2009 to 2017 for PL) while controlling for season and groups (JM, JF, AM, PF, LF) on biochemical analytes (marginal mean, lower 95% CI to higher 95% CI) from YFPs.

Analyte	Poyang Lake (PL)	Time PL^a^	Tian-E-Zhou Oxbow (TZO)	Time TZO^a^	Location	Seasonal Sidak Comparison^c^
		Coef., *P* value		Coef., *P* value	*P* value^b^	
ALT (U/l)	31.7, 28.5–35.3	−0.0476, 0.01	39.1, 34.2 to 44.8	NSD	0.03	S < W & F
AST (U/l)	197.2, 185.1–209.4	−5.973, 0.002	218.9, 203.7–234.1	NSD	0.05	S < W & F
AST/ALT	6.3, 5.7–6.9	NSD	5.5, 4.8–6.3	NSD	NSD	NSD
GGT (U/l)	38.0, 35.6–40.6	−0.0302, 0.008-	40.3, 36.9–44.0	NSD	NSD	NSD
ALP (U/l)	130.2, 112.0–151.3	0.0532, 0.03	170.0, 141.1–204.8	NSD	NSD	NSD
TBA (μmol/l)	4.16, 3.47–5.0	NSD	8.0, 6.39–10.0	NSD	< 0.0001	F < W
T-BILI (μmol/l)	3.37, 2.85–3.94	NSD	2.71 2.15–3.34	0.0562, 0.001	NSD	NSD
D-BILI (μmol/l)	0.69, 0.52–0.87	NSD	1.01, 0.77–1.3	0.0514, < 0.001	0.06	S & Sm & F < W
I-BILI (μmol/l)	1.99, 1.16–2.47	NSD	1.42, 1.09–1.9	0.0604, 0.01	NSD	NSD
TP (g/l)	76.4, 74.7–78.0	NSD	70.3, 68.5–72.3	NSD	<0.0001	NSD
ALB (g/l)	43.8, 41.9–45.7	NSD	50.6, 48.1–53.2	−0.762, 0.039	0.0002	W < S
GLB (g/l)	32.8, 30.7–34.9	NSD	18.4, 15.7–21.2	NSD	<0.0001	S < W
ALB/GLB	1.34, 1.16–1.55	NSD	3.53, 2.91–4.30	NSD	<0.0001	W < S
BUN (mmol/l)	17.6, 16.8–18.5	NSD	18.5, 17.4–19.6	NSD	NSD	NSD
UA (μmol/l)	48.2, 41.1–56.4	−0.1024, <0.001	41.3, 34.0–50.2	NSD	NSD	S < W
TC (mmol/l)	5.5, 5.11–5.83	−0.1997, 0.01	6.40, 5.97–6.82	NSD	0.004	NSD
TG (mmol/l)	4.26, 3.44–5.27	NSD	2.37, 1.84–3.04	NSD	0.002	NSD
HDL-C (mmol/l)	2.36, 2.21–2.50	−0.232, <0.001	3.21, 3.03–3.39	NSD	<0.0001	S & F < W
LDL-C (mmol/l)	1.75, 1.50–2.04	0.1128, <0.001	1.83, 1.50–2.22	−0.1442, <0.001	NSD	W < F
HDL-C/LDL-C	5.81, 4.65–7.26	−2.175, <0.001	8.07, 6.15–10.59	NSD	NSD	S & F < W
CK (U/l)	90.3, 75.1–106.9	−1.064, <0.001	156.9, 132.0–183.9	NSD	0.0001	S < W
LDH (U/l)	224.0, 203.8–246.2	0.067, <0.001	233.8, 207.5–263.3	NSD	NSD	F & W < S
AMS (U/l)	15.6, 11.6–20.1	8.577, < 0.001	4.57, 2.11–8.0	NSD	0.0005	F < W
Glucose (mmol/l)	7.64, 7.37–7.91	NSD	7.99, 7.6–8.38	NSD	NSD	W < F & S
CO_2_ (mmol/l)	21.6, 20.3–22.8	NSD	26.3, 24.2–28.5	NSD	0.002	NSD
Cr (μmol/l)	75.3, 71.0–80.0	NSD	78.1, 72.5–84.2	NSD	NSD	F < S
K^+^ (mmol/l)	4.4, 4.2–4.5	NSD	4.0, 3.8–4.2	−0.012, 0.08	0.032	NSD
Na^+^ (mmol/l)	156.7, 155.8–157.6	NSD	152.8, 151.7–154.0	−0.344, 0.003	<0.0001	NSD
Cl^−^ (mmol/l)	108.7, 107.8–109.4	NSD	108.3 107.3–109.4	−0.3345, 0.005	NSD	W & S < F
Ca^2+^ (mmol/l)	2.56, 2.53–2.60	−0.0135, 0.008	2.47, 2.42–2.51	NSD	0.003	NSD
PO_4_	1.58, 2.45–1.70	0.0838, <0.001	1.28, 1.12–1.43	NSD	0.013	W & S < F
Mg^2+^ (mmol/l)	2.26, 2.15–2.37	0.0584, 0.003	2.06, 1.95–2.18	NSD	0.05	NSD
Fe^2+^ (μmol/l)	27.6, 23.2–32.3	NSD	33.9, 27.4–41.1	NSD	NSD	NSD

NSD: Not significantly different (*P* > 0.05). JM = juvenile male, JF = juvenile female, AM = adult male, PF = pregnant female, LF = lactating female.

^a^Results of linear regression using time as the independent variable for analyte data (dependent variable, across all years and animal groups) calculated separately for each location.

^b^Significance (*P* ≤ 0.05) determined by location (TZ vs. PL) specific marginal mean (controlled for variance due to group and season) post hoc sidak comparison of the respective biochemical analytes.

^c^Significance (*P* ≤ 0.05) determined for season (W = winter, S = spring, Sm = summer, F = fall) specific marginal mean (controlled for variance due to group and location) post hoc sidak comparison of the respective biochemical analytes. Only significantly different seasons are shown.

### Comparison of biochemical parameters from YFP between each habitat (TZO vs. PL)

Significant increases in the hepatic enzymes (ALT, AST,) and the hepatobiliary system (TBA, D-BILI) [approximate significance, *P* = 0.06] were detected in the animals located at TZO (Table 3). However, PL animals had increased (*P* < 0.0001) TP, decreased (*P* = 0.0002) ALB and almost doubled (*P* < 0.0001) GLB compared to TZO (Table 3). For TZO animals, TC was increased (*P* = 0.004) while TG was almost half (*P* = 0.002) than those observed in PL, but HDL-c was increased (*P* < 0.0001, Table 3). The AMS was three times higher (*P* = 0.0005) in PL animals, while CK in TZO YFP was almost double (*P* = 0.0001) than PL animals. Finally, CO_2_ was elevated (*P* = 0.002) while K^+^, Na^+^ Ca^2+^ PO_4_ and Mg^2+^ were significantly decreased in TZO animals (Table 3).

### Biochemical parameter changes across the years for animals living in PL and TZO YFPs, respectively

#### Poyang Lake

Significant decreases in the liver related analytes ALT, AST and GGT were detected. No other significant changes were noted for liver related biochemical values (Table 3). The lipid profile indicated a significant decrease in TC, HDL-c and HDL-c/LDL-c, while a significant increase in LDL-c was detected (Table 3). Both the UA and CK concentrations decreased significantly, while LDH and AMS both significantly increased. For electrolytes, Ca^+^ decreased while Mg^2+^ and PO_4_ increased (Table 3).

#### Tian-E-Zhou Oxbow

Significant changes in the hepatic system were characterized by an increase in T-BILI, D-BILI and I-BILI. Similarly, decreases (*P* = 0.04) in ALB were noted, however, no changes in liver enzymes over time were detected. A decrease (*P* < 0.001) in LDL-c concentration was also noted. Finally, a significant decrease in the Na^+^ and Cl^-^ were noted, with a near significant decrease in K^+^ (*P* = 0.08; Table 3).

### Comparisons of marginal mean biochemical parameters between groups (JM, JF, AM, PF, LF) combined across both locations

The YFPs showed significant decreases in ALP, CK and increased in TP with age (*P* < 0.05, Fig. 1). The Ca^2+^ was non-significantly higher in juveniles (JM, JF) versus AM and significantly higher than PF (Fig. 1). Inter sex differences within adults could not be measured directly since we did not sample any non-pregnant, non-lactating adult females and both pregnancy and lactation are known to affect multiple analytes in killer whales (Robeck and Nollens, 2012). However, within the juveniles, only GLU was significantly increased in JM compared to JF. The GGT was significantly greater in adult males compared to JF, PF and LF (Fig. 1). Pregnant YFP had significantly reduced AST, AST/ALT, CO_2_, Ca^2+^ compared to JF, and significantly increased GLB, BUN and TG compared to JM, AM and both juveniles and AM, respectively. The LF had significantly increased HDL-c and K^+^ compared to PF and AM, respectively (Fig. 1).

### Comparisons of biochemical parameters in YFP between each location (TZO vs. PL) within each group (JM, JF, AM, P, L)

#### Juvenile males

**Figure 2:**
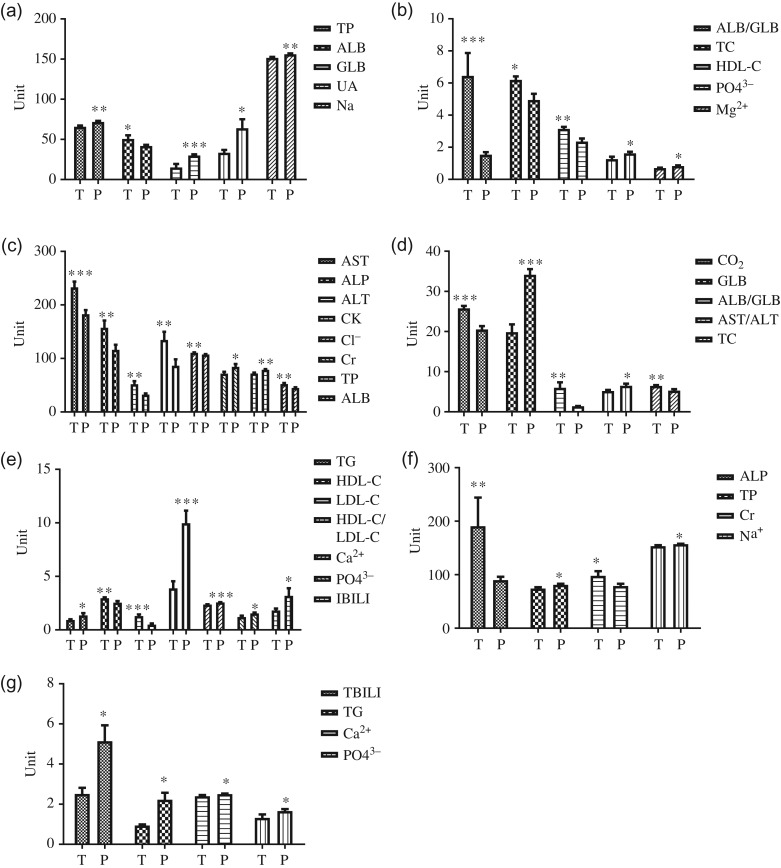
Overall comparison of the biochemical parameters between the Tian-E-Zhou Oxbow (T) and Poyang Lake (P) YFPs. The figures (**a**, **b**) compared the biochemical parameters between the T and P juvenile males. The figures (**c**, **d**, **e**) indicate biochemical differences in the adult males between T and P animals. The biochemical differences in pregnant groups are indicated by figures (**f**, **g**) between the T and P animals. The significant differences between the two populations are indicated by asterisk at ^*^P < 0.05, **P < 0.01 and ***P < 0.001.

The overall results of biochemical parameters are summarized in (Fig. 2). Juvenile male (JM) dwelling in the PL showed significantly higher serum level of GLB, Mg^2+^, Na^+^, PO4^3-^, TP and UA. In TZO YFPs, serum ALB, ALB/GLB, HDL-C and TC were significantly higher.

#### Adult males

For AM living in the TZO, the hepatic enzymes (ALP, ALT, AST), lipid profile (TC, HDL-c, LDL-c) and other biochemical parameters (ALB, ALB/GLB, CK, Cl^−^, CO_2_) were significantly higher than the adult males (AM) of PL YFPs. On the other hand, AM living in the PL showed significantly higher serum levels of hepatic enzymes (I-BILI, AST/ALT), lipid profile (TG, HDL-c/LDL-c) and other biochemical parameters (Ca^2+^, Cr, GLB, PO4^3^^−^, TP) as shown in (Fig. 2).

#### Pregnant females

The same as observed in other groups, serum levels of TP, PO4^3−^, Ca^2+^, Na^+^ and TG along with TIBILI were significantly higher in the PL YFPs. Only serum ALP and Cr were significantly higher in the TZO individuals (Fig. 2).

## Discussion

### Biochemical differences between locations and across time

#### Liver parameters

The hepatobiliary system of the TZO YFPs appeared to be under increased activation as demonstrated by a significant increase in bilirubin (T-BILI, D-BILI and I-BILI) over time and significant increases in liver associated analytes including ALP, AST, ALP, T-BILI, TBA and decreased TP compared to animals located at PL. In addition, during the entire study period, animals at PL had significant decreases in ALT, AST and GGT. Bile acids and bilirubin are typically used as indicators of liver clearance and can be elevated during states of parenchymal liver disease and biliary obstruction (Limdi and Hyde, 2003). Similarly, AST and ALT are typically increased during hepatocellular injury due to multiple clinical etiologies such as viral hepatitis, toxic hepatitis, cholestatic hepatitis and chronic active hepatitis (Rosalki and Mcintyre, 1999; Limdi and Hyde, 2003). Alternatively, significantly higher serum ALT, AST and ALP paired with the significantly increased serum cholesterol concentrations in the TZO animals suggests steatosis (Bayard *et al.*, 2006). Total protein was reduced, albumin, which is produced by the liver and decreased in liver dysfunction, was increased for animals at TZO verses PL. The exact cause for elevated albumin in TZO needs further investigations. Complicating this determination is the fact that cetaceans are known to have heavy reserve capacity for hepatic albumin production. In addition, dehydration can elevate albumin production. Therefore, these factors limit the use of albumin as an early indicator of hepatic disorders (Bossart *et al.*, 2001). The GLB were half and ALB/GLB was double those at TZO verse PL and were the cause of the observed decrease in TP.

Water quality in the TZO has recently been reported as being of reduced quality when compared to PL (Nabi *et al.*, 2017a). We are aware (our unpublished work) that the TZO is primarily influenced by agricultural non-point pollution, natural input, poultry excrement and decayed organic matter pollutants. In addition to pesticides, poultry discharge from local poultry farms have been entering the TZO for over 20 years. Poultry discharge is known to possible contain viruses, bacteria, parasites, veterinary pharmaceuticals and heavy metals (EPA, 1998; University of Iowa and Iowa State Study Group, 2002; Boxall *et al.*, 2003; Wei *et al.*, 2010). All of these toxic chemicals or biologics are metabolized by or can directly affect the liver and may account for the increase in hepatic associated enzymes. Consequently, the significant changes observed in the hepatobiliary system of TZO over time are of important concern.

#### Lipid profile

In Poyang Lake YFPs, we observed a significant increase in LDL-C, and a significant decrease in TC, HDL-C and HDL-C/LDL-C as compared to TZO, while a significant decrease in LDL-C in the TZO YFPs over time. (Table 3). Variation in the YFPs lipid profile may be affected by changing fisheries resources, overall animal nutritional health, habitat utilization and the reproductive cycle (Pethybridge *et al.*, 2011a, b; Kim *et al.*, 2012; Nabi *et al.*, 2017b). In cetaceans, such as bottlenose dolphins (*Tursiops truncates*), beluga whales (*Delphinapterus leucas*) and pantropical spotted dolphins (*Stenella attenuata*), the effects of diet on the lipid profile has been reported (Asper *et al.*, 1990; Cook *et al.*, 1990; St. Aubin *et al.*, 2013). A significant variation in the lipid profile in response to habitat dynamics may indicated changing prey availability between the two locations (Miller *et al.*, 2012). In the PL, there are ample of evidences that overfishing (Chen *et al.*, 2002; Wei *et al.*, 2007; Li, 2008), illegal fishing, using illegal fish gears (Wang, 2009; Schelle, 2010), and removal of large numbers of fish and shrimps by sand mining machine (Yu *et al.*, 2001), are reducing the availability or diversity of prey for the YFPs. Furthermore, water pollution, acoustic pollution and habitat degradation (Warner, 2008; Chen *et al.*, 2009) have already threatened several fish species within this environment (Chen *et al.*, 2004; Hvistendahl, 2008). Similarly, in the TZO, the increasing population of YFPs (Wang, 2015) combined with possible fish mortality and morbidity by various toxic chemical pollutants in the reserve (Nabi *et al.*, 2017a) deplete or change the availability of prey for the YFPs.

#### Other enzymes

The significantly higher levels of serum CK and non-significantly higher concentration of LDH in TZO YFPs could be due to rhabdomyolysis linked to capture stress as these animals are occasionally exposed to chasing during capture (Williams and Pulley, 1983; Gasper and Gilchrist, 2005). Despite using the same capture method for both populations, animals in the TZO were apparently more active when compared to the PL (Nabi *et al.*, 2017a). Therefore, the overall significantly higher CK level in the adult male of TZO might be due to the hyper-muscular activities (Paola *et al.*, 2007).

#### Electrolytes

The significant decrease in serum levels of Na^+^, K^+^ and Cl^−^ in TZO as compared to PL, and a significant increase in the serum levels of Mg^2+^ and PO4^3^^−^ while decrease in the Ca^+^ levels of PL YFPs over time may reflect endocrine and gastrointestinal conditions (Bossart *et al.*, 2001) of YFPs in response to a changing habitat (Sun *et al.*, 2012; Dong, 2013). Overall, the electrolytes (Ca^2+^, PO4^3^^−^, Mg^2+^, Na^+^) were significantly higher in the PL YFPs compared to TZO YFPs where only Cl^-^ was significantly higher. In addition to dehydration, hyperaldosteronism, liver and renal dysfunctions elevate Na^+^ levels in cetacean (Bossart *et al.*, 2001). Similarly, dehydration, renal disease, hypoadrenocorticism and primary hyperparathyroidism increase the serum Ca^2+^ concentration (Bossart *et al.*, 2001). The Mg^2+^, PO4^3^^−^ and Cl^−^ levels are affected by renal problems. However, PO4^3^^−^ is also affected by rhabdomyolysis, dietary phosphorus excess, osteolytic bone disease, and hypoparathyroidism and hypercalcemia with normal glomerular filtration (Bossart *et al.*, 2001). While differentiating clinical conditions from normal homeostatic variations in response to dietary, or environmental differences would require further diagnostics, these significant changes are worth noting.

### Biochemical profile changes during maturation and reproductive state

Serum ALP was significantly higher in the juveniles compared to the adults. While Ca^2+^ was also increased in juveniles compared to adults with significant differences found when compared to PF. Both ALP, and Ca^2+^ have been reported to be increased in juveniles of multiple cetaceans including killer whales, bottlenose dolphins and beluga (Andersen, 1968; St Aubin *et al.*, 2001; Venn-Watson *et al.*, 2011; Nollens *et al.*, 2018). Higher ALP and Ca^2+^ concentrations in young animals is generally associated with active bone growth (Andersen, 1968; Kovacs, 2001) and has been used to indicate physical maturity in other mammals including finless porpoises (Andersen, 1968). Increased CK in young animals was also observed in killer whales, bottlenose dolphins and pigs (Thorén-Tolling, 1982; Venn-Watson *et al.*, 2007; Nollens *et al.*, 2018). This increase during growth may be an indicator of muscle development in both species, but without isoenzyme identification a similar phenomenon occurring in YFP is only unknown.

Similar to what was observed in killer whales (Robeck and Nollens, 2013), Pregnant YFP had significantly decreased AST, AST/ALT ratio and increased GLB, TG. Liver enzyme decreases were attributed to volume expansion during pregnancy in killer whales and mare (Harvey *et al.*, 2005). However, contrary to our results in YFP whereby BUN increased, this volume expansions also results in increased renal clearance and decreased BUN (Robeck and Nollens, 2013). Therefore, volume expansion may not be as significant in the relatively short gestation of the YFP (~<12 month) as compared to the killer whale (17.5 month, Robeck *et al.*, 2015). Since BUN is considered metabolic waste of protein metabolism, the increase during YFP gestation may simply reflect increase food intake during gestation. The increase in TG were also observed in the killer whale (Robeck and Nollens, 2013) and in humans and horses this change has been attributed to estrogen mediated increase in Very Low Density Lipoprotein (VLDL) (Alvarez *et al.*, 1996; Harvey *et al.*, 2005).

Only HDL-C was elevated during lactation, while this change hints at the well-documented changes in lipid mobilization during lactation in most mammals (Koopman *et al.*, 2002; Struntz *et al.*, 2004), changes in TG, and TC post-partum are commonly observed in other species (Struntz *et al.*, 2004; Robeck and Nollens, 2013). This lack of observed changes in TG and TC during lactation may indicate differences in YFP physiology or is most likely due to extremely small sample set of animals from which detecting significant deviations from the other groups was not possible.

### Seasonality in biochemical parameters

While complete evaluation of seasonal changes could not be conducted due to the lack of sampling in the summer months, multiple parameters exhibited significant differences between the remaining three seasons. Serum creatinine in the YFPs showed seasonality with significantly higher levels in spring vs fall suggesting the effects of nutrition as observed in captive and wild bottlenose dolphins (Terasawa *et al.*, 2002; Hall *et al.*, 2007). We are aware (unpublished work) that in both populations of YFPs, prey availability is higher in the spring and summer and therefore these changes could reflect higher intake of prey. Furthermore, increased creatinine concentration during spring and summer months has been attributed to seasonal alteration in muscle mass in bottlenose dolphins (Hall *et al.*, 2007; Macchi *et al.*, 2011). In addition, higher concentrations of creatinine have been associated with increased and prolonged physical exertion (Gasper and Gilchrist, 2005) and this increase in spring for the YFP may be due to the increased socially driven physical activity associated with seasonality of reproduction in both males and females. For YFP, a seasonal spring increase in GLU was also observed and may also indicate metabolic changes in response to increased physical activity. Seasonal changes in activity can also be associated with changes in both demand and types of prey availability. For example, in the polar bear, concentrations of GLU correlate with seasonal changes in the percentage of dietary protein and fat (Bossart *et al.*, 2001). The synthesis of ALB is directly associated with protein intake (Hall *et al.*, 2007), therefore, higher serum ALB and ALB/GLB in spring provides additional support for increased prey consumption during the spring. The increased serum UA concentrations observed in winter for the YFP has also been reported in bottlenose dolphin and could be due to seasonal variation in the nutrient composition of their diet (Hall *et al.*, 2007) as a diet rich in purines can cause hyperuricemia (Villegas *et al.*, 2012). Similarly, seasonal variations in the lipid profile, electrolytes and hepatobiliary parameters of YFPs could be due to changes in the water components, changes in body condition, water temperature or quality, diet, photoperiod and other factors (Domingo-Roura *et al.*, 2001; Terasawa *et al.*, 2002; Sergent *et al.*, 2004; Norman *et al.*, 2013).

## Conclusions and future recommendations

In summary, our findings provide indirect evidence for potential changes in fisheries resources in both populations as indicated by a significantly different and changing lipid profiles within and between the two populations. If the cause is from a declining fisheries resources in the TZO, it may be due to pesticides induce fish mortality, morbidity and low productivity. To regulate the Oxbow, fisheries resources and the total population numbers should be closely managed. Similarly, in the PL, illegal fishing and the use of non-selective fishing gears should be strictly avoided. While significant age specific differences were noted, all could be attributed to normal physiologic changes with age that have also been observed in other cetacean species. Across age groups, however, both the populations in general and TZO YFPs specifically showed hepatic dysfunction as indicated by higher hepatobiliary parameters which might be in response to various pollutants such as pesticides and poultry. The TZO also showed differing concentrations of various electrolytes as compared to PL which suggest gastrointestinal and endocrine changes whose etiology requires further investigation. Our findings suggest that YFPs in the TZO have not been removed from the negative effects of escalating anthropogenic activities especially, pesticides pollutions. Therefore, to conserve the YFPs in the TZO, special and immediate attention is required to improve the water quality and control the discharge from agriculture runoffs. In addition, future efforts at quantifying current concentrations of PCBs and other hydrocarbon bioaccumulations in fat and blood in both populations should be prioritized.
